# Systematic evaluation of signal-to-noise ratio in variant detection from single cell genome multiple displacement amplification and exome sequencing

**DOI:** 10.1186/s12864-018-5063-5

**Published:** 2018-09-17

**Authors:** Anita T. Simonsen, Marcus C. Hansen, Eigil Kjeldsen, Peter L. Møller, Johnny J. Hindkjær, Peter Hokland, Anni Aggerholm

**Affiliations:** 1 Department of Hematology, Aarhus University Hospital, Aarhus, Denmark; 2 AAGAARD Skejby Fertility Clinic, Aarhus, Denmark

**Keywords:** Single cell sequencing, Sparse cell sequencing, Allele dropout, Signal-to-noise ratio, Whole exome sequencing, NGS

## Abstract

**Background:**

The current literature on single cell genomic analyses on the DNA level is conflicting regarding requirements for cell quality, amplification success rates, allelic dropouts and resolution, lacking a systematic comparison of multiple cell input down to the single cell. We hypothesized that such a correlation assay would provide an approach to address the latter issues, utilizing the leukemic cell line OCI-AML3 with a known set of genetic aberrations.

**Results:**

By analyzing single and multiple cell replicates (2 to 50 cells) purified by micromanipulation and serial dilution we stringently assessed the signal-to-noise ratio (SNR) from single as well as a discrete number of cells based on a multiple displacement amplification method, with whole exome sequencing as signal readout. In this setting, known OCI-AML3 mutations as well as large copy number alterations could be identified, adding to the current knowledge of cytogenetic status. The presence of DNMT3A R882C, NPM1 W288 fs and NRAS Q61L was consistent, in spite of uneven allelic read depths. In contrast, at the level of single cells, we observed that one-third to half of all variants were not reproduced in the replicate sample, and this allelic mismatch displayed an exponential function of cell input. Large signature duplications were discernible from 5 cells, whereas deletions were visible down to the single cell. Thus, even under highly optimized conditions, single cell whole genome amplification and interpretation must be taken with considerable caution, given that allelic change is frequent and displays low SNR. Allelic noise is rapidly alleviated with increased cell input, and the SNR is doubled from 2 to 50 cells.

**Conclusions:**

In conclusion, we demonstrate noisy allele distributions, when analyzing genetic aberrations within single cells relative to multiple cells. Based on the presented data we recommend that single cell analyses should include replicate cell dilution assays for a given setup for relative assessment of procedure-specific SNR to ensure that the resolution supports the specific hypotheses.

**Electronic supplementary material:**

The online version of this article (10.1186/s12864-018-5063-5) contains supplementary material, which is available to authorized users.

## Background

Detailed characterization of sparse cancer subpopulations, and even single cells, by next generation sequencing (NGS) modalities is rapidly gaining momentum, potentially adding valuable information on the biology underlying neoplasia and progression. These approaches hold important bearings for future personalized therapy in both mono- and oligoclonal disease entities, where the cancerous stem cell population is the ultimate therapeutic target. Arguably, single cell analysis is not a new phenomenon, and has to some extent been described by both cytogenetics and flow cytometry, although NGS offers unprecedented possibilities for high throughput, informational content and scalable sensitivity. In parallel with the development of single cell research whole exome sequencing (WES) has progressed to a mature method in cancer research and laboratory diagnostics with high informational value, though the extent of its use is still driven by cost versus diagnostic or research benefit.

Until fairly recently, the use of NGS in single cell analysis has been limited, partly due to the requirement of a relatively large amount of input material. This problem can now be circumvented by whole genome amplification (WGA), but reports on the quality and consistency of these techniques are still lacking. Currently, there are three major WGA-methods; the multiple displacement amplification (MDA), degenerate-oligonucleotide-primed PCR (DOP-PCR) and multiple annealing and looping-based amplification cycles (MALBAC). A comparison of the three, according to variant detection, has recently been performed [1, 2], without a clear conclusion on which is the most optimal approach. Importantly, several sources of contributing noise are introduced during WGA, such as decreased coverage uniformity, regional loss of coverage, polymerase errors, allelic imbalance and allele dropout. The last term is not consistently defined throughout different studies [3]. Added to these different results is the fact that, while single cell resolution is the ultimate feat for characterization of discrete subclonal contribution to leukemogenesis and potential targeted therapy, allelic substitutions or dropouts will invariably occur with increasing frequency when fewer and fewer cells are analyzed, questioning the value of apparently somatic observations. In some aspects, the present literature remains ambiguous and incoherent regarding the quality of single cell DNA genomic analysis with large variation in the reported allelic dropout rates [3], with values as high as 40–50% reported using MDA [4, 5], which is currently the most commonly used method [3]. In spite of these attempts to map the allelic dropout rate, one piece is evidently missing: The evaluation of single cell performance and resolution in NGS, compared to a low number of multiple cells, which is the focus here. In consideration of this gap, we decided to revisit the concept, evaluating and suggesting a simple, but essential, strategy for quality assessment of single and sparse cell assays based on allelic read depths.

Our study employs a well-characterized leukemia cell line, OCI-AML3 [6], and one of the most established methods for WGA: MDA. We set out to investigate and formalize a signal-to-noise ratio (SNR), and to elaborate on the components of this, correlate single and sparse cell allelic read depths, allele frequency dispersion and distributions, and to characterize allele dropout as a clear continuous function of cell input. Often, as is the case with OCI-AML3, no clear normal control sample exists, which otherwise can be implemented for somatic variant calling and copy number analysis – the latter through read depth ratios. However, as we also set to demonstrate, allelic imbalance directly reflects large chromosomal copy number variation in these unpaired samples. It is our assumption that such general analyses can be utilized regardless of amplification or capture methods, and thus an assessment on the performance of the individual WGA methods is beyond the focus of this paper.

## Methods

### Cell culturing

The OCI-AML3 cell line was obtained from Deutsche Sammlung von Mikroorganismen und Zellkulturen (DSMZ, Braunschweig, Germany) and cultured in RPMI-1640 medium (Invitrogen, Thermo Fisher Scientific, CA, USA) with 5% FCS and antibiotics (penicillin, streptomycin). The culture was physically isolated from other cultures throughout the incubation. Harvesting was performed in the exponential growth phase.

### Micromanipulation, whole genome amplification and genotyping

The cultured OCI-AML3 cells were dispensed in RPMI/PBS-medium and replicates of single, 2, 5 and 10 cells were subsequently separated by micromanipulation under a dissection microscope, using a fine glass pipette. The 25 and 50 cell sample replicates were created using serial dilutions. The aliquots of 1, 2, 5, 10, 25 and 50 cells were manipulated into 200 μL PCR tubes in a total volume of 4 μL PBS and prepared for WGA (REPLI-g Single Cell Kit, Qiagen, Hilden, Germany). Genomic characterization of WGA DNA included 21 short tandem repeat loci (STR, PowerPlex 21 System, Promega, WI, USA), also employed in forensic genome identification, of which 18 loci were known to be heterozygous in OCI-AML3, fragment analysis of known *NPM1*^W288fs^ mutation [7] and qPCR of the *DNMT3A*^R882C^ mutation [8]. Median allele dropout was calculated by comparing replicate sample loci from STR. The described molecular analyses were performed in six single cell replicates and in triplicates for 2–50 cell input.

### Whole exome sequencing, sequencing processing and variant analysis

For exome sequencing 1, 2, 5, 25 and 50-cell replication assays were prepared as described above. Sample replicates likewise underwent WGA with subsequent WES on the Illumina HiSeq 2500 platform (Aros Applied Biotechnology, Eurofins, Aarhus, DK (5–50 cell assays), and Rigshospitalet, Center for Genomic Medicine, Copenhagen, DK (1 and 2-cell assays)). Two micrograms from each WGA sample were submitted to library preparation performed by the sequencing provider using Nextera Rapid Capture 37 Mb kit (Illumina, San Diego, CA, USA). Sequencing of the ten amplified samples aimed at a theoretical mean depth of coverage of 90. Fastq files of 100 bp paired-end reads were aligned and processed using BWA and Picard, and variant calling were performed with Genome Analysis ToolKit (GATK 3.6, Broad institute, Cambridge, MA, USA. See Additional file 1: Table S1 for full details). Phred-scaled genotype likelihoods was derived directly from GATK variant output (see GATK User Guide, software.broadinstitute.org). Downstream annotation of single nucleotide variants (SNVs) was performed in VariFant (varifant.com, Aarhus, DK) and variant analyses were performed with Mathematica (Wolfram Research, Champaign, IL, USA). Allelic mismatch was computed by comparing replicate assays for each cell subsets, i.e. from distinct biological input of equal cell concentration. Copy number alterations (CNA) were resolved by allelic imbalance in chromosomal variant allele frequency assessment (read depth threshold ≥30) and kernel density estimation, using Gaussian smoothing and Silverman’s rule for bandwidth selection. Calculation of read depth ratios from paired control was not possible.

### Cytogenetics

Chromosome preparations were examined on bulk OCI-AML3 material according to standard laboratory protocols after 24-color karyotyping with the 24XCyte kit as described previously [9]. Cytogenetic and molecular cytogenetics analyses were done according to ISCN 2013 principles [10].

## Results

### Allele dropout as a function of cell input

**Fig. 1:**
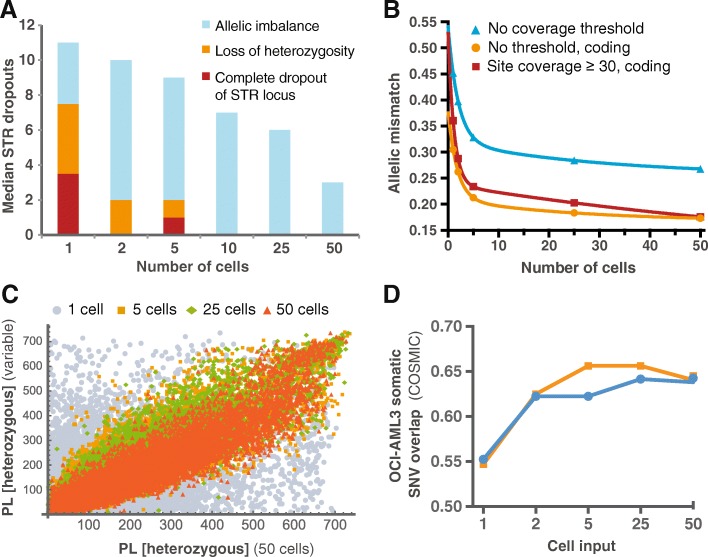
Loci dropouts, allelic mismatch and read depth variation from sparse cell assays. The correlation between molecular marker dropout and low cell input is evident from microsatellite genotyping (STR) based on 21 different loci compared to bulk DNA from OCI-AML3 (**a**). This dropout was manifested by a complete absence of a specific marker loci (A, red), unambiguous loss of heterozygosity (**a**, orange) or partial dropout (**a**, blue), represented as imbalance in heterozygosity. Intersection of variants from replicate genome amplification and exome sequencing (**b**) shows allelic mismatch between duplicate assays, i.e. degree of reproducibility, as an approximately exponential function of cell input. Inclusion of all raw GATK-passed variants led to an allelic mismatch of approximately 50% (**b**, blue line). The proper inclusion of only coding variants decreased this number to one-third (B, orange), whereas setting a coverage threshold (red) did not additionally improve the result. Allelic read depths are highly influenced by the number of cells used for amplification as demonstrated here by the relative comparison of phred-scaled likelihood of each being called heterozygous (*P* = 10^(-PL/10)^) to one replicate of the 50-cell assays (**c**, 0.51 < *ρ*_Spearman_ < 0.84) – directly a result of read depth variation of both reference and alternate alleles. Comparison of the single nucleotide variant (SNV) sets from 1 to 50 cells against the reported somatic SNVs in the COSMIC database (286 SNVs, Cell Lines Project v85, OCI-AML3) revealed only a minor decrease in variant overlap (**d**, blue) as cell input decreased. This was also the case, when focusing on the more confident subset of 31 SNVs marked as *reported previously* in COSMIC (**d**, orange)

We initially set out to determine the extent of allelic change between replicate assays as marker for the reproducibility of WGA. Microsatellite genotyping of STR consistently showed a high frequency of allele dropout in single and low cell numbers from the evaluation of partial or complete loss of heterozygosity (LoH) and full loci dropout (Fig. 1a). Median dropouts from these assays, employing micro-manipulated cells in the 1–10 cell assays, ranged from 3 to 11 of 21 loci, inversely correlating with cell number. Complete LoH of a locus or full locus dropout was absent from 10 input cells and above. Next, we assessed the WGA performance by allelic reproducibility by means of WES, comparing replicate cell sample variant sets. A mean of 9.4 × 10^7^ reads was achieved (8.3–12.7 × 10^7^) with 99.3% of the sequences mapped to human reference genome (GRCh37), thus yielding alignment efficacy comparable to bulk exome sequencing [11]. In spite of this, allelic mismatch measured from duplicate sequencing remained a significant problem, where close to 50% of the SNVs were not detected in the second single cell replicate (Fig. 1b, based on GATK-passed variants), while decreasing exponentially to one-third at 5 cells. Restriction of variants to coding regions alone alleviated the high discrepancy between single cell replicates to one-third, thus noise is exacerbated outside targeted regions. Mismatch was defined as LoH, change of the variant allele or total dropout, while the latter was found negligible. These results strongly indicate that the frequent loss of one allele in the replicate originated from large variations in variant read depth by uneven amplification or amplification errors. This notion is supported by correlation analysis (0.51 < *ρ*_Spearman_ < 0.84) of variant genotype likelihood of the 50 cell-assay to its replicate and lower input counterparts (Fig. 1c), and from the fact that low input assays contained a higher number of detected variants (Additional file 2: Figure S1). A very low genotype correlation was evident when comparing 50 cells with amplified genomes from single cells. This effect was exacerbated, when single and two-cell replicates were compared (*data not shown*).

### Detections of known cell line mutations from variable cell input

Despite the inconsistencies in variant read depths, the ability to detect known cell line mutations was generally consistent by all laboratory modalities. Thus, detection of *DNMT3A*^R882C^ mutation by qPCR was found in all replicates from 2-cell input and above. Moreover, the *NPM1* type A somatic mutation, leading to frameshift p.W288fs, was detected in all fragment analyses. All sequence subset replicates confirmed the mutations, inspected in IGV (Broad Institute), with a broad range of coverage (Median 81× (8–315×)) and allele depths (Additional file 3: Figure S2). Also, the NRAS^Q61L^ mutation [12], was detected in all sequencing result sets. Mean variant allele frequencies (VAF) were 41.5% (25.9–75%) and 44.5% (31.3–57.1%) in *DNMT3A* and *NPM1*, respectively. These observations were supported by the relatively high overlap and consistency, when comparing SNVs from the cell replicates and proposed somatic variants in the COSMIC Cell Lines Project (55–66%). Only a minor decrease in variant overlap was found in the single cell replicate (Fig. 1d).

### Detection of large stretches of allelic imbalance

**Fig. 2:**
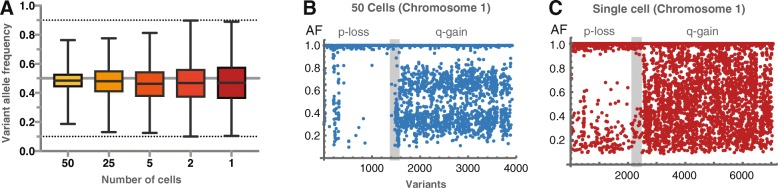
Increased variability in low cell input. Increased dispersion of heterozygous allele frequencies is observed with low cell input (**a**). The range cannot be determined exactly in one and two cell assays due to threshold cut-offs, but extend from at least 10 to 90%, and are heavily affected by noise. The effect of variable read depths is observed from chromosomal allele frequencies, which makes copy gains, resolved from approximately 50 cells (**b**), difficult to detect, when approaching the single cell level (**c**). This effect is also demonstrated by normalized probability density comparison of trisomy 8 as shown in the supplement (Additional file 4: Figure S3)

**Fig. 3:**
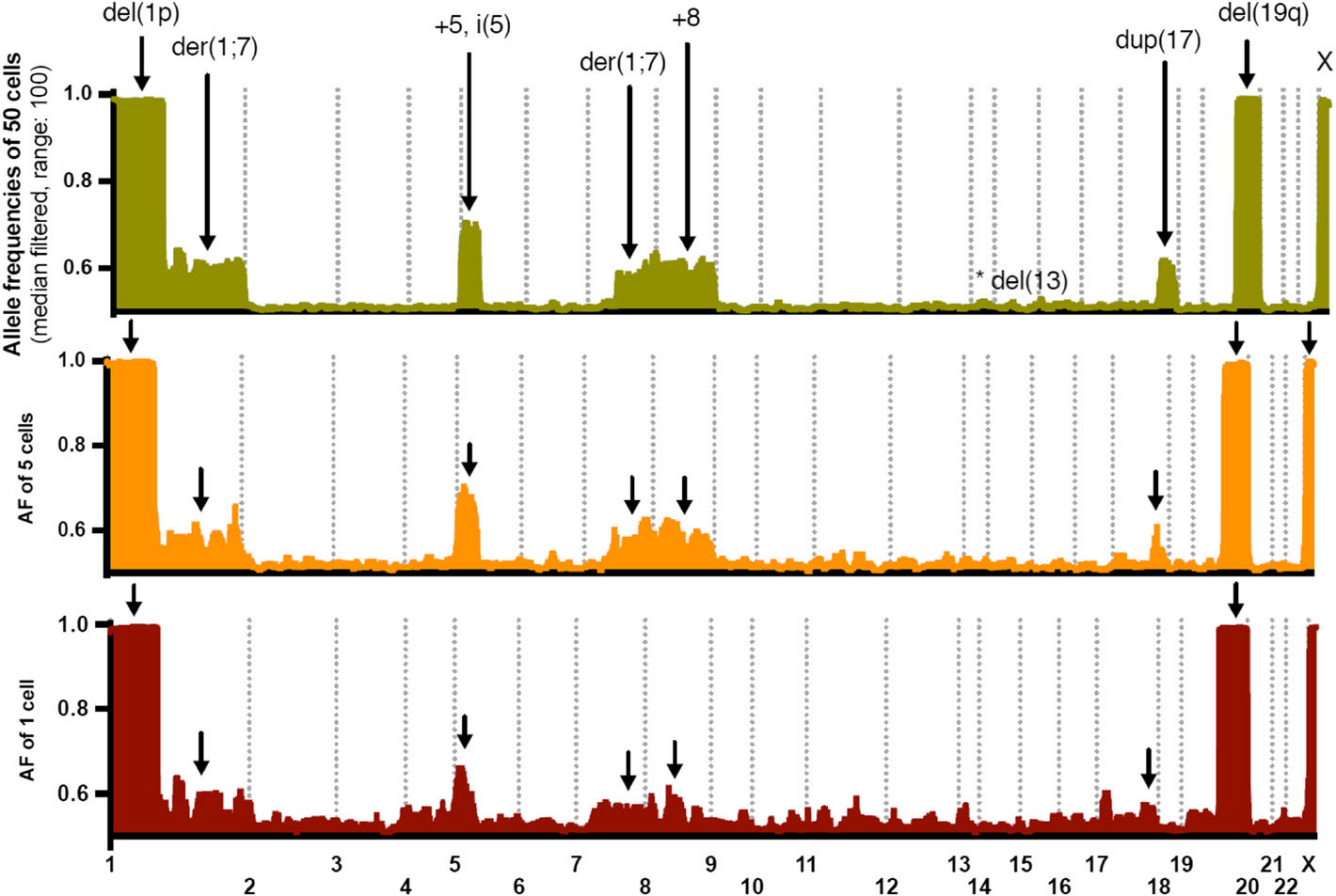
Deterioration of allele frequencies (AF) and copy number signals by decreased cell input. Each plot reflects median filtered (neighborhood range of 100 variants) AF of all chromosomes from duplicate assays. AF shifts as a result of copy number changes are readily identified for a high number of input cells (50), whereas this pattern is not readily discernible from the duplicate single cell assay (2 and 25 cell plots are found in the Additional file 5: Figure S4). The noise reduction also resulted in a false-negative del(13) comprising less than 100 variants (Additional file 6: Figure S5)

Next, we turned to copy number assessment, since OCI-AML3 displays a range of partial or complete gains or losses. In order to address the question of whether CNA can be resolved at low cell input, we took advantage of allelic imbalance to detect CNA. While other methods exist, such as evaluating log-scaled read depth ratios, the method described here does not directly require a paired control sample but is compared with the results from conventional cancer cytogenetics. As stated, the difference in read depths, allele dropouts and highly variable allele frequency values (Fig. 2a) have a profound impact on single cell and sparse cell resolution. This is also the case for detection of allelic imbalance: Whereas both partial loss and gain could be resolved from the 50-cell assay on chromosome 1 (Fig. 2b), only the p-arm loss at could be visually resolved from the single cell assays (Fig. 2c). More sophisticated, the kernel density estimation technique enabled the detection of large duplications from 50 down to 5 or 2 cells (Additional file 4: Figure S3). Noise reduction, such as median filtering, was necessary for the general detection and relative comparison of CNAs (Fig. 3 and Additional file 5: Figure S4), at the cost of sensitivity as exemplified by false negative loss on chromosome 13 (Fig. 3 and Additional file 6: Figure S5). Collectively, these data show that the detection of copy gains in single cells is very difficult, heavily affected by noisy allele distributions, but was – in this case – directly visible from 2 to 5 cells and upwards.

### Independent confirmation of copy number variation

**Fig. 4:**
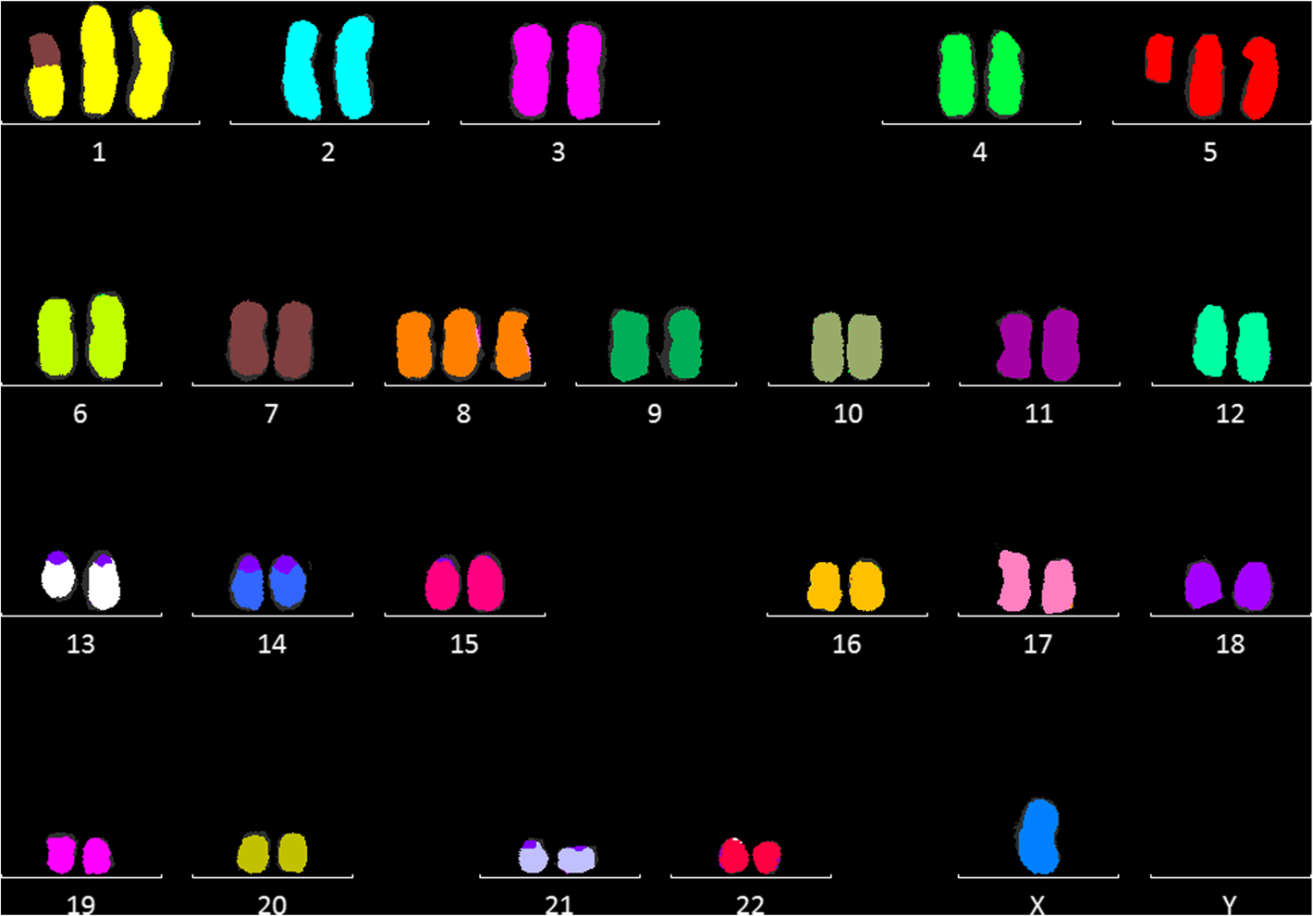
Color karyotyping of the OCI-AML3 cell line leading to the ISCN karyotype 48,X,-Y,+ 1,der(1;7)(p11;q22),+ 5,i(5) (p10),+ 8,del(13) (q13q21),dup(17)(q21q25) [6]. The only discrepancy between exome sequencing and resulting cytogenetic profile was large deletion of 19q, classified as a copy neutral LoH and detectable down to the single cell

Since WES is still at an experimental level with regards to CNA detection allele frequency shifts from sequencing were compared to 24-color karyotyping and literature in order to independently confirm deduced copy numbers (Table 1 & Fig. 4). Whereas translocation involving chromosome 1 and 7 was observed (der(1;7)(p11;q22)), the originally described der(1)t(1;18)(p11;q11) was, evidently, not. Thus, corroborated by partial duplication of the affected chromosomes from WES allele frequency analysis the published karyotype may be previously inadequately described. Also, del(19q), not described in the literature, was consistently found in single cells and greater, but absent in the updated color karyotype 48,X,-Y,+ 1,der(1;7)(p11;q22),+ 5,i(5) (p10),+ 8,del(13) (q13q21),dup(17)(q21q25) [6]. As such, this may point to a copy neutral aberration on chromosome 19, which, in contrast to the previous translocation, may be a clonal newcomer. Summarizing, WES analysis of CNAs from a low cell number by means of allele frequency shifts matched the conventional color karyotype. Also, LoH, with copy or copy neutral loss, is unambiguously manifested down to the single cell.

**Table 1:** Comparison of detected cytogenetic aberrations

Analysis	dup(1q)	del(1p)	der(1)t(1;18) (p11;q11)	der(1;7) (p11;q22)	+i(5p)	+ 8	del(13)	dup(17)	del(19q)
Quentmeier & al.	+	–	+	–	+	+	+	+	–
24-color karyotyping	+	–	–	+	+	+	+	+	–
Exome sequencing	+^a^	+	–	+^b^	+^c^	+	+	+^a^	+

^a^ Gains were detected from 5 cells and up, whereas losses were detected down to single cells. ^b^ The translocation involves a copy-gain and is thus matched by whole exome sequencing allele frequency analysis. ^c^ Observed as gain

### Generalizing the concept of allelic noise in sequencing

**Fig. 5:**
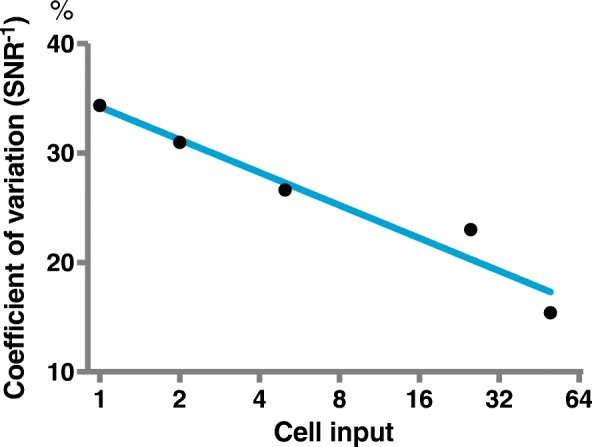
Coefficient of variation or signal-to-noise (SNR) ratio as an unbiased measure of heterozygous allelic dispersion and quality. As allele dropout is ambiguously defined, we suggest the addition of SNR of heterozygous variants to describe the quality of single or sparse cell data. It is defined as the level of true signal, here represented as heterozygous alleles of one-half, compared to the level of the background noise or dispersion as defined as the standard deviation of the allele frequencies, i.e. the reciprocal of the coefficient of variation

We have previously observed that the standard deviation of heterozygous allele frequencies from whole exome bulk sequencing is rather constant between samples of approximately same read coverage, where the frequencies assume Gaussian distributions (*not shown*). By selecting chromosomes not effected by allelic imbalance from CNAs, such as chromosome 2 and 3 used here, this characteristic formed a more objective evaluation of allelic resolution. Defined as the level of true signal compared to the level of the background noise, SNR can be expressed as μ/σ, where the reciprocal is recognized as the *coefficient of variation (CV)*. We find that the SNR from 50 cells is more than doubled compared to the single cell replicates (Additional file 7: Table S2) and approximates a log-linear function (Fig. 5). Also, the heterozygous allele frequency distributions from 50-cell replicates were found to approximate the normal distribution (Anderson-Darling test), as is the case with high quality bulk sequencing (*not shown*).

## Discussion

Recent advances in hardware designs, such as microfluidic systems, have generated a steep rise in papers dealing with detailed characterization of cancers down to the single cell level. While such studies, aimed at defining intratumoral heterogeneity and evolution, have contributed significantly, there has been no consensus on how to perform and report quality control, be it single cells used or pooling results from multiple single cells. In fact, a recent exhaustive review found that the term allelic dropout is ambiguously defined [3], which is why we introduce the addition of SNR, or the reciprocal coefficient of variation, as a more unambiguous estimation of quality for individual low-input samples. One major problem in quality control is that the difference from bulk analysis to the single cell level lacks intermediate comparison to sparse cell analysis. Performing serial dilutions for calibration purposes is one of the oldest tricks of the trade, which has more or less escaped notice as for relative assessment of single cells with regards to sensitivity and specificity. Thus, at the present time, the literature has conflicting elements on the extent of allelic dropout, which cast doubt on the usability of the underlying methods and generated data from single cell studies, when addressing biological questions. We hypothesized that a controlled setting, involving cell dilution, could address the questions regarding the extent of allelic dropout or allelic mismatch, SNR and varying read depths in a given setup, which is currently lacking in the literature.

Several technologies hold the promise of accomplishing single cell purification, such as Fluidigm integrated fluidic circuit (IFC) systems (South San Francisco, CA, USA) and fluorescence-activated cell sorting, as we implement in parallel studies. C1 part of the Fluidigm product line has previously been shown to have a suboptimal capture efficacy for medium IFCs (PN 101–2711 A1 White Paper, Fluidigm, [13–16]), and for this reason, we opted to use micromanipulation, which is operator-controlled with immediate visual quality assurance of the captured cells and numbers.

Apart from cell capture, other variables include the cell source, amplification method, the workflow concerning WES library preparation and sequencing depth. Each of these factors will contribute to the distribution of noise but can be characterized collectively, when keeping cell input as the independent variable and other factors fixed. Here, we opted for working with MDA using a well-known hematopoietic cell line, since it should theoretically provide an input population with the intraclonal heterogeneity of primary cancer cell isolates brought to a minimum. Though in vitro development of molecular lesions in cell lines is a theoretical inherent problem, which could interfere with this experimental design, it is a minor issue here given that all experimentation was performed with the same batch of cells.

While we demonstrate compatible results between STR analysis and WES, the resolution from the latter is much higher. Whereas, genotyping covered 21 loci, here amounting to more than four hundred individual sites to be analyzed from the replicate dilution assays, each exome analysis offers additional data points by approximately three orders of magnitude from coding variants alone. We thus suggest the presented approach to explore questions relating to noisy distributions and allele dropout in single and sparse cell analysis in order to describe the underlying biology more confidently. Despite highly comparable percentage of genomic reference mapping between single cell sequencing and expected from previous bulk exome sequencing, large variations in allelic read depth and allele frequencies were observed. Still, we resolved large reproducible stretches of LoH on chromosome 1 and 19 down to single cells, which has not been described previously. These particular losses do not appear by conventional cytogenetics, and thus may represent a copy-neutral LoH. Furthermore, the lack of t(1;18) is ascribed inadequately characterization of the original important study, which also pinpointed the archetypical *NPM1* mutation in myeloid malignancies. The positive outcome here is that even in very few cells, as exemplified by five-cell assay here, it is possible to clearly detect trisomies and deletions. Likewise, mutation calling is deemed rather robust in replicates, although allele dropout may occur in few cell assays. The clear advantage of these methodologies to characterize rare, heterogeneous subpopulations, or even to potentially profile the discrete biology of each single cell, is evident.

## Conclusions

Based on the data presented, it may be argued that single cell DNA WES is still highly problematic by its variable nature, but, more importantly, peer researchers must be able to evaluate published results on a solid basis and calls for improved QC. We conclude that, while the possibility to detect known mutations from single cells is apparently consistent, the detection of unknown somatic mutations will be affected by allele read depth and frequency variation and calls for caution. The lesson learned is that read frequency distributions and SNR, in relative comparison to multiple cells, must be included in every study. This is valid regardless of the capture or amplification method in order to assess the feasibility of detecting biological diversity – in contrast to technical variability.

## Additional files

Additional file 1: **Table S1.** Exome sequencing alignment and variant calling commands. (PDF 45 kb)

Additional file 2: **Figure S1.** The number of GATK-passed variants increase as cell input decreases. (PDF 39 kb)

Additional file 3: **Figure S2.** Variability of allele depths. The known driver mutations are shown along with another focus variant MET T1010I of unknown relevance. (PDF 34 kb)

Additional file 4: **Figure S3.** Kernel density estimates of allele frequencies enables the detection of trisomy down to two cells. Variant alleles are normally a bimodal distribution, whereas trisomies display a trimodal distribution as shown for chromosome 8. The Gaussian function was used as kernel and bandwidth selection was based on Silverman’s rule ((4σ^5/3n)^1/5). The functions have been scaled to same maximum (at VAF = 1) for relative comparison. As a result, the Y-axis is unitless. (PDF 117 kb)

Additional file 5: **Figure S4.** Deterioration of allele frequencies (AF) and copy number signals by decreased cell input. The plots show the median filtered (range of neighborhood was set to 100 variants) allele frequencies (AF). All AFs (replicate mean) below 0.5 was mirrored to the equal distance above for an improved signal-to-noise ratio. While the signal deteriorates for copy gains with smaller cell input, the signal of the deletions does not. (PDF 429 kb)

Additional file 6: **Figure S5.** Scatter plot of OCI-AML3 chr 13 showing medial q-arm deletion and possible small distal deletion of both 50-cell assay replicates. Scatter plots of replicate allele frequencies reveal known partial chr 13 deletion. Short stretches of loss of heterozygosity, relative to the total number of called variants or size of the chromosome, does not severely affect the distribution of heterozygous variant allele frequencies. This is apparent from both Q-Q plots, frequency comparison to an unrelated bulk sequencing sample and test for normality (Anderson-Darling). (PDF 374 kb)

Additional file 7: **Table S2.** Noise assessment in sparse cell sequencing analysis. The table shows and increase in signal-to-noise as cell input increases. Only the 50-cell assays were found to approximate a normal distribution (Anderson-Darling). (PDF 65 kb)

## Abbreviations

AF: Allele frequencies
CNA: Copy number variation
LoH: Loss of heterozygosity
MDA: Multiple displacement amplification
NGS: Next generation sequencing
SNR: Signal-to-noise ratio
STR: Short tandem repeat loci
WES: Whole exome sequencing
WGA: Whole genome amplification
