# Supplementary material for: Systematic evaluation of signal-to-noise ratio in variant detection from single cell genome multiple displacement amplification and exome sequencing
Source: BMC Genomics. 2018 Sep 17;19:681. doi: 10.1186/s12864-018-5063-5 (PMC6142419; doi:10.1186/s12864-018-5063-5)
Supplement: Supplementary file 1 — Table S1. Exome sequencing alignment and variant calling commands. (PDF 45 kb) [file 12864_2018_5063_MOESM1_ESM.pdf]

ADDITIONAL FILE

Table S1

```
#!/bin/sh
```

Aligning to reference genome

```
bwa mem -M -R ”@RG\tID:group1\tSM:$FILE_NAME\tPL:illumina\tLB:lib1\tPU:unit1” \
    -t 15 $P2HG19/hg19gatkidx $FILE_PATH_IN$FILE_NAME_F $FILE_PATH_IN$FILE_NAME_R \
    > $FILE_PATH$FILE_NAME.sam
```

Sorting aligned reads

```
java -Xmx4g -jar SortSam.jar INPUT=$FILE_PATH$FILE_NAME.sam \
    OUTPUT=$FILE_PATH$FILE_NAME.sorted.sam SORT_ORDER=coordinate
```

Deduplication of reads

```
java -Xmx4g -jar MarkDuplicates.jar INPUT=$FILE_PATH$FILE_NAME.sorted.sam \
    OUTPUT=$FILE_PATH$FILE_NAME.sorted.dedup.bam \
    METRICS_FILE=$FILE_PATH$FILE_NAME.metrics.txt
```

Building index

```
java -Xmx4g -jar BuildBamIndex.jar INPUT=$FILE_PATH$FILE_NAME.sorted.dedup.bam
```

Using GATK 3.6 for variant workflow

```
P2GATK=Tools/GenomeAnalysisTK-3.6
```

Indel realignment

```
java -Xmx4g -jar GenomeAnalysisTK.jar -T RealignerTargetCreator -nt 8 \
    -R $P2HG19/ucsc.hg19.fasta \
    -I $FILE_PATH$FILE_NAME.sorted.dedup.bam \
    -known $P2HG19/Mills_and_1000G_gold_standard.indels.hg19.sites.vcf \
    -o $FILE_PATH$FILE_NAME.sorted.dedup.intervals.list

java -Xmx4g -jar GenomeAnalysisTK.jar -T IndelRealigner \
    -R $P2HG19/ucsc.hg19.fasta \
    -I $FILE_PATH$FILE_NAME.sorted.dedup.bam \
    -targetIntervals $FILE_PATH$FILE_NAME.sorted.dedup.intervals.list \
    -o $FILE_PATH$FILE_NAME.sorted.dedup.realign.bam \
    -known $P2HG19/Mills_and_1000G_gold_standard.indels.hg19.sites.vcf
```

Recalibration of quality scores

```
java -Xmx4g -jar GenomeAnalysisTK.jar -T BaseRecalibrator -nct 8 \
    -R $P2HG19/ucsc.hg19.fasta \
    -I $FILE_PATH$FILE_NAME.sorted.dedup.realign.bam \
    -knownSites $P2HG19/dbsnp_138.hg19.vcf \
    -knownSites $P2HG19/Mills_and_1000G_gold_standard.indels.hg19.sites.vcf \
    -o $FILE_PATH$FILE_NAME.sorted.dedup.realign.1stpass.table

java -Xmx4g -jar GenomeAnalysisTK.jar -T BaseRecalibrator -nct 8 \
    -BQSR $FILE_PATH$FILE_NAME.sorted.dedup.realign.1stpass.table \
    -R $P2HG19/ucsc.hg19.fasta \
    -I $FILE_PATH$FILE_NAME.sorted.dedup.realign.bam \
    -knownSites $P2HG19/dbsnp_138.hg19.vcf \
    -knownSites $P2HG19/Mills_and_1000G_gold_standard.indels.hg19.sites.vcf \
    -o $FILE_PATH$FILE_NAME.sorted.dedup.realign.2ndpass.table

java -Xmx4g -jar GenomeAnalysisTK.jar \
    -R $P2HG19/ucsc.hg19.fasta \
    -T PrintReads -BQSR $FILE_PATH$FILE_NAME.sorted.dedup.realign.2ndpass.table \
    -I $FILE_PATH$FILE_NAME.sorted.dedup.realign.bam \
    -o $FILE_PATH$FILE_NAME.sorted.dedup.realign.recal.bam
```

Variant calling

```
java -Xmx4g -jar $P2GATK/GenomeAnalysisTK.jar -l INFO \
    -R $P2HG19/ucsc.hg19.fasta -T UnifiedGenotyper \
    -I $FILE_PATH$FILE_NAME.sorted.dedup.realign.recal.bam \
    -o $FILE_PATH$FILE_NAME.vcf -nct 8 \
    -stand_call_conf 30.0 \
    -stand_emit_conf 10.0 \
    --dbsnp $P2HG19/dbsnp_138.hg19.vcf

java -Xmx4g -jar GenomeAnalysisTK.jar \
    -T VariantRecalibrator \
    -R $P2HG19/ucsc.hg19.fasta \
    -input $FILE_PATH$FILE_NAME.vcf \
-resource:hapmap,known=false,training=true,truth=true,prior=15.0 $P2HG19/hapmap_3.3.hg19.sites.vcf \
-resource:omni,known=false,training=true,truth=false,prior=12.0 $P2HG19/1000G_omni2.5.hg19.sites.vcf \
-resource:dbsnp,known=true,training=false,truth=false,prior=6.0 $P2HG19/dbsnp_138.hg19.vcf \
    -an QD -an HaplotypeScore -an MQRankSum -an ReadPosRankSum -an FS -an MQ -mode SNP \
    -recalFile $FILE_PATH$FILE_NAME.recalibrated.vcf \
    -tranchesFile $FILE_PATH$FILE_NAME.tranches \
    -rscriptFile $FILE_PATH$FILE_NAME.plots.R

java -Xmx4g -jar $P2GATK/GenomeAnalysisTK.jar \
  -T ApplyRecalibration \
  -R $P2HG19/ucsc.hg19.fasta \
  -input $FILE_PATH$FILE_NAME.vcf \
  --ts_filter_level 99.0 \
  -tranchesFile $FILE_PATH$FILE_NAME.tranches \
  -recalFile $FILE_PATH$FILE_NAME.recalibrated.vcf -mode SNP \
  -o $FILE_PATH$FILE_NAME.recalibrated.filtered.vcf
```
