# Supplementary figures and images for: Systematic evaluation of signal-to-noise ratio in variant detection from single cell genome multiple displacement amplification and exome sequencing
Source: BMC Genomics. 2018 Sep 17;19:681. doi: 10.1186/s12864-018-5063-5 (PMC6142419; doi:10.1186/s12864-018-5063-5)

FIGURE S1

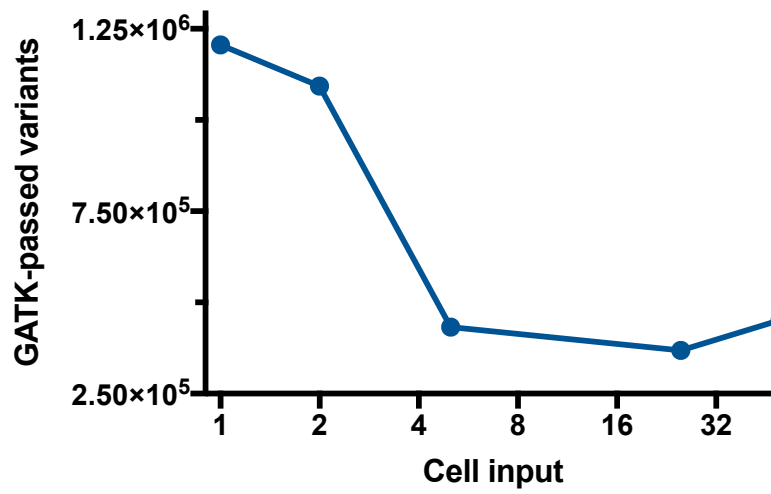

Supplement: Supplementary file 2 — Figure S1. The number of GATK-passed variants increase as cell input decreases. (PDF 39 kb) [file 12864_2018_5063_MOESM2_ESM.pdf]

FIGURE S2

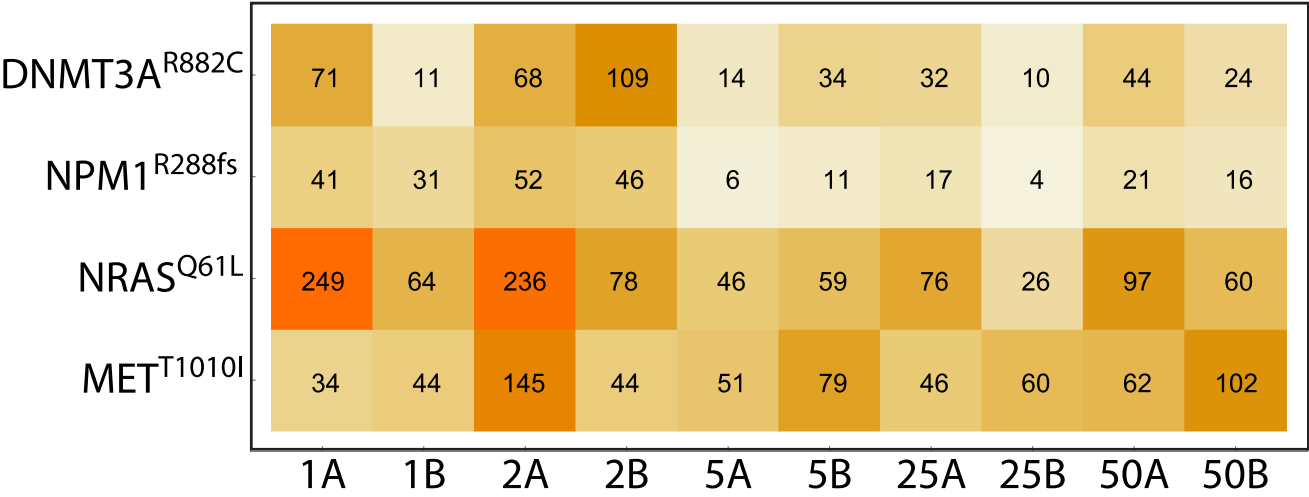

Supplement: Supplementary file 3 — Figure S2. Variability of allele depths. The known driver mutations are shown along with another focus variant MET T1010I of unknown relevance. (PDF 34 kb) [file 12864_2018_5063_MOESM3_ESM.pdf]

FIGURE S3

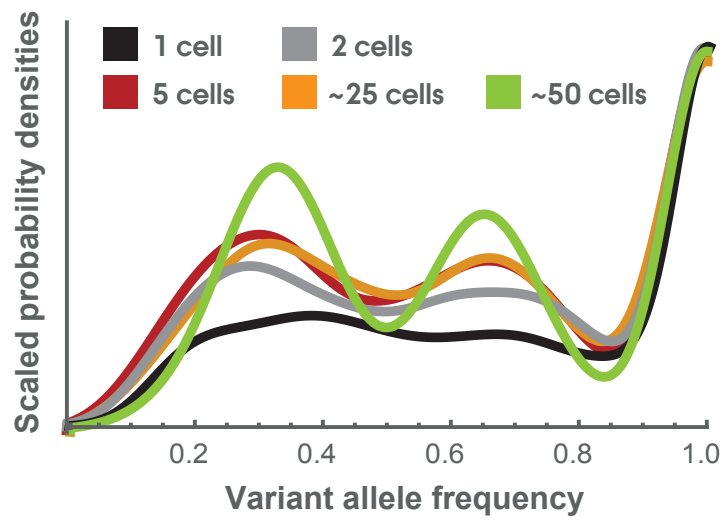

Supplement: Supplementary file 4 — Figure S3. Kernel density estimates of allele frequencies enables the detection of trisomy down to two cells. Variant alleles are normally a bimodal distribution, whereas trisomies display a trimodal distribution as shown for chromosome 8. The Gaussian function was used as kernel and bandwidth selection was based on Silverman’s rule ((4σ^5/3n)^1/5). The functions have been scaled to same maximum (at VAF = 1) for relative comparison. As a result, the Y-axis is unitless. (PDF 117 kb) [file 12864_2018_5063_MOESM4_ESM.pdf]

FIGURE S4)

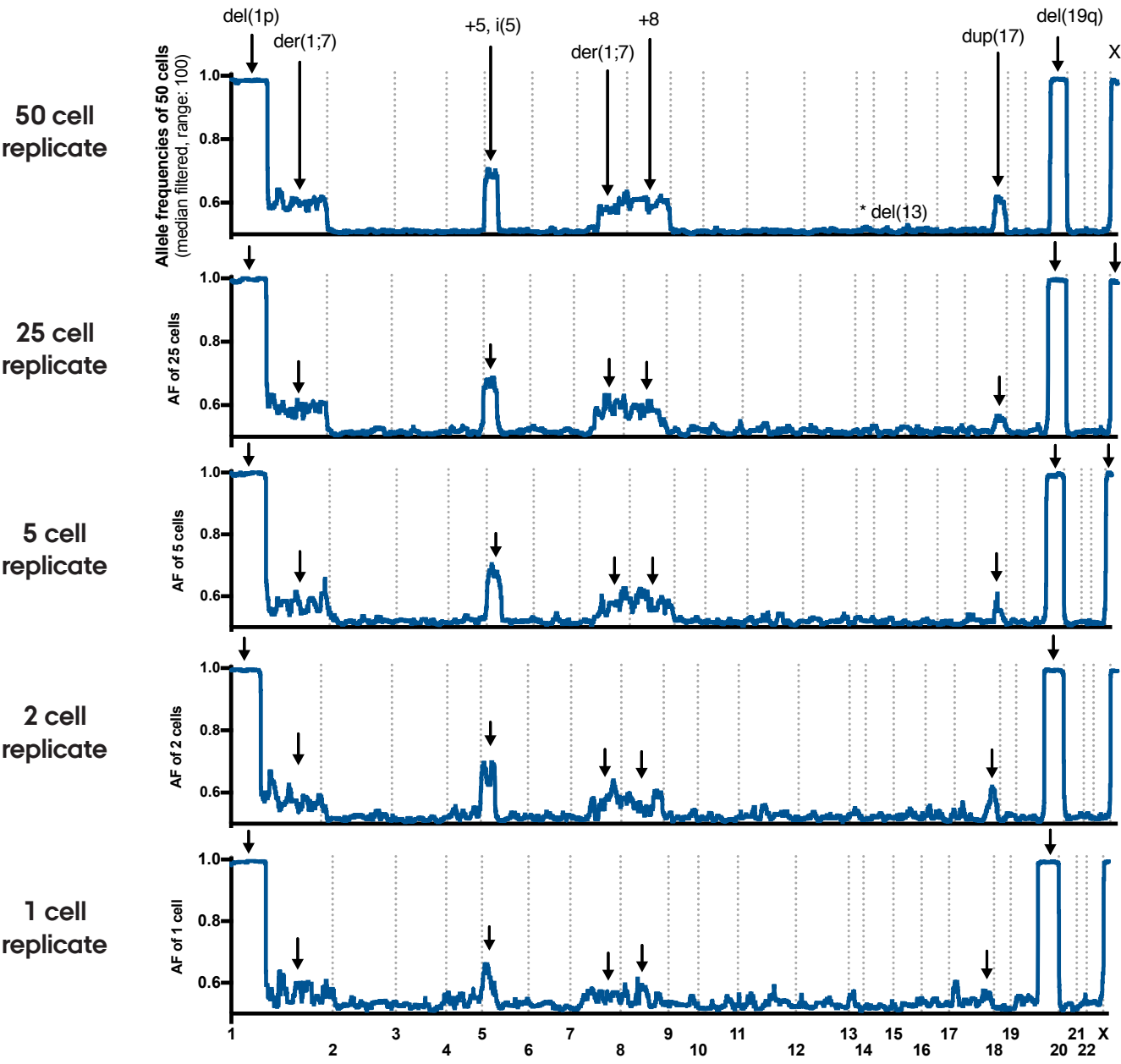

Supplement: Supplementary file 5 — Figure S4. Deterioration of allele frequencies (AF) and copy number signals by decreased cell input. The plots show the median filtered (range of neighborhood was set to 100 variants) allele frequencies (AF). All AFs (replicate mean) below 0.5 was mirrored to the equal distance above for an improved signal-to-noise ratio. While the signal deteriorates for copy gains with smaller cell input, the signal of the deletions does not. (PDF 429 kb) [file 12864_2018_5063_MOESM5_ESM.pdf]

FIGURE S5)

Replicate 1

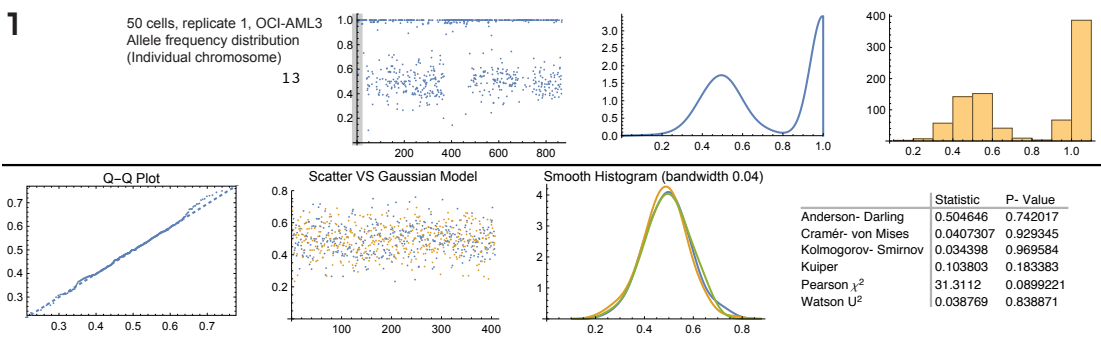

Replicate 2

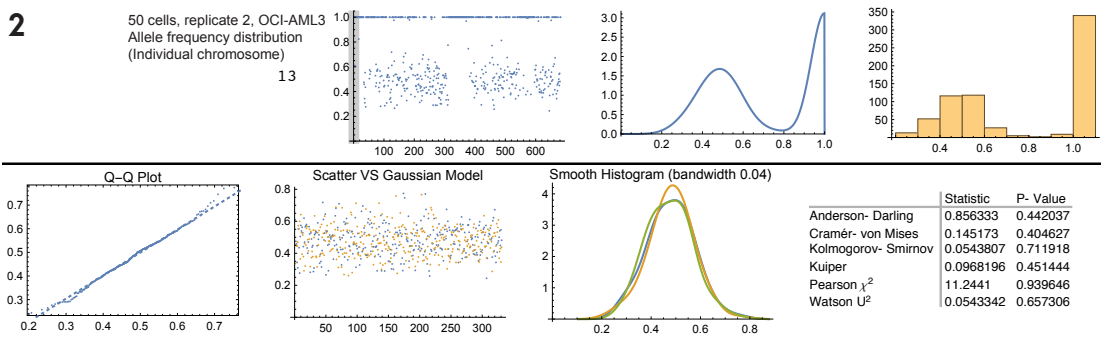

Supplement: Supplementary file 6 — Figure S5. Scatter plot of OCI-AML3 chr 13 showing medial q-arm deletion and possible small distal deletion of both 50-cell assay replicates. Scatter plots of replicate allele frequencies reveal known partial chr 13 deletion. Short stretches of loss of heterozygosity, relative to the total number of called variants or size of the chromosome, does not severely affect the distribution of heterozygous variant allele frequencies. This is apparent from both Q-Q plots, frequency comparison to an unrelated bulk sequencing sample and test for normality (Anderson-Darling). (PDF 374 kb) [file 12864_2018_5063_MOESM6_ESM.pdf]
