# Supplementary material for: Systematic evaluation of signal-to-noise ratio in variant detection from single cell genome multiple displacement amplification and exome sequencing
Source: BMC Genomics. 2018 Sep 17;19:681. doi: 10.1186/s12864-018-5063-5 (PMC6142419; doi:10.1186/s12864-018-5063-5)
Supplement: Supplementary file 7 — Table S2. Noise assessment in sparse cell sequencing analysis. The table shows and increase in signal-to-noise as cell input increases. Only the 50-cell assays were found to approximate a normal distribution (Anderson-Darling). (PDF 65 kb) [file 12864_2018_5063_MOESM7_ESM.pdf]

Table S2)

**Cell replicate**	**SNR (μ/σ)**	**SD (σ)**	**Mean (μ)**	**Normal distribution***
50	6.49	0.075	0.49	Yes (p=0.11)
25	4.35	0.11	0.48	No (0.02)
5	3.75	0.12	0.46	No (0.01)
2	3.23	0.14	0.47	No (<0.0001)
1	2.91	0.16	0.47	No (<0.0001)

*Anderson-Darling test was used on heterozygous variants not affected by copy number changes
